# Editorial: Surgical Selection of Sublobar Resection and Scope of Lymph Node Dissection for Early-Stage Lung Cancer

**DOI:** 10.3389/fsurg.2022.915220

**Published:** 2022-05-11

**Authors:** Jiajing Sun, Chengchu Zhu, Jianfei Shen

**Affiliations:** Taizhou Hospital, Zhejiang University, Taizhou, China

**Keywords:** NSCLC, lymphadenectomy decision, segmentectomy, lymph node, sublobar resection

**Editorial on the Research Topic**

***Surgical selection of sublobar resection and scope of lymph node dissection for early-stage lung cancer***

The standard treatment for non‒small cell lung cancer (NSCLC) includes lobectomy, pneumonectomy, and sublobar resection. In selecting the surgical method, doctors need a safer, less invasive method that can be done more rapidly and provide the most suitable type of resection at different stages of NSCLC. Sublobar resection (including segmental resection and wedge resection) can treat smaller lesions and offers better recovery of postoperative pulmonary function. In early-stage lung cancer in patients who are physically fit and have good lung function, there seems to be no difference in perioperative mortality and morbidity rates for lobar or sublobar resection (1). Therefore, sublobar resection is often selected as the surgical method for early-stage lung cancer.

Lymph node dissection, especially more extensive regional dissection, is thought to improve survival rates in patients with early-stage lung cancer (2). It is often necessary to combine lymph node dissection with sublobar resection in patients with early-stage lung cancer to estimate the progress of the tumor and the stage of cancer. Researchers on the topic “strategies of lymph node dissection during sublobar research for early-stage lung cancer” highlight the need for preoperative evaluation and intraoperative lymph node dissection in patients with early-stage lung cancer. They also provide a snapshot of the status of the research and the ongoing research on sublobar resection.

The preoperative evaluation of benign and malignant pulmonary nodules in early-stage cancer plays a significant role in the selection of surgical method and prognosis of patients. Common evaluation methods include imaging, gene detection, and tumor-related marker detection. Ye et al. (3) conducted a prospective study of 125 preoperative patients with NSCLC with nodules of 1 cm or less. Their analysis showed that patients with pulmonary nodules of 10 mm or less detected by circulating genetically abnormal cells (CACs) before operation could effectively diagnose lung cancer and help doctors choose the appropriate treatment. Imaging is indispensable in the preoperative evaluation of pulmonary nodules, but it is not sufficient for the total analysis. Therefore, Wei et al. (4) combined CEA, CYFRA21-1, and NSE tumor markers with imaging to evaluate benign and malignant pulmonary nodules. They found that the sensitivity of tumor markers combined with imaging in classifying lung cancers was significantly better, at 90%, than other diagnostic methods. The NSE marker combined with imaging was more advantageous in the diagnosis of small cell lung cancer.

In addition, multivariable logistic regression models with backward selection based on the Akaike information criterion were used to identify independent predictors. Xia et al. (5) retrospectively studied 763 patients with pulmonary nodules who underwent lobectomy or sublobar resection. They summarized a set of prediction models for the preoperative evaluation of benign and malignant pulmonary nodules, which was used to estimate the possibility of lung cancer in nodules requiring surgical treatment. The discriminatory value of the prediction model was better than the commonly used Mayo or Brock model. The high accuracy of the evaluation model could reduce unnecessary surgery for benign nodules, improve the diagnosis and treatment of malignant nodules, and prolong the survival of patients.

Preoperative chemotherapy is commonly used in patients with advanced NSCLC. However, interestingly, for early-stage (stage I) small cell lung cancer, Ye et al. (6) recommended routine preoperative chemotherapy. They conducted a cohort study of 477 patients with limited-stage small cell lung cancer (LS-SCLC). The patients were divided into two groups according to whether they received preoperative chemotherapy or not. The overall survival (OS) and cancer-specific survival (CSS) rates were recorded. Using propensity score matching, the inverse probability of treatment weight, and the superposition weight analysis, they concluded that surgery plus chemotherapy significantly improved the median OS rate of LS-SCLC patients compared with surgery alone.

The lymph node metastasis of lung cancer is an important reason for the disease’s high mortality rate, so lymph node dissection and exploration in sublobar resection of early NSCLC is very important. Gossot et al. (7) proposed that lymph node resection is the key link in sublobar resection. Only when lymph node dissection is included does the survival rate after segmental resection become equivalent to that of lobectomy. Therefore, the authors recommended using the rapid immunohistochemical method to quickly detect tumor metastases in lymph nodes during operation, this method can help in decisions about whether to expand the scope of the resection, and it can ensure the same outcomes as with lobectomy, which are radical resection, free margins, and full clearance of the lymph nodes. The number of lymph nodes removed was also related to the prognosis for patients. The only prognostic factor seen in the multivariate analysis of the 5-year survival rate was the number of lymph nodes resected. Coincidentally, in the study of Liu et al. (8), it was mentioned that when more than six lymph nodes were examined, the diagnostic rate of lymph node metastasis was significantly higher and the 5-year survival rate was also significantly higher. Compared with the more commonly used wedge resection, anatomical resection is more advantageous in removing the number of lymph nodes. However, there are still different opinions about the choice of sublobar resection and lymph node dissection. Zhang et al. (9) retrospectively analyzed 1644 patients (422 with adenocarcinoma in situ [AIS] and 1,222 with minimally invasive adenocarcinoma [MIA]). It was concluded that for AIS/MIA patients, sublobar resection, especially wedge resection without lymph node dissection, resulted in no significant difference in the prognosis compared with other surgical methods with a larger resection range. Therefore, we conclude that wedge resection may be a better choice for patients with ground glass nodules of AIS and MIA and that anatomical resection for disease in more than six lymph nodes can provide a better prognosis for early lung cancer with more extensive invasion.
